# Impact of Pals1 on Expression and Localization of Transporters Belonging to the Solute Carrier Family

**DOI:** 10.3389/fmolb.2022.792829

**Published:** 2022-02-16

**Authors:** Carmen Berghaus, Ann-Christin Groh, Davorka Breljak, Giuliano Ciarimboli, Ivan Sabolić, Hermann Pavenstädt, Thomas Weide

**Affiliations:** ^1^ University Hospital of Münster (UKM), Internal Medicine D (MedD), Münster, Germany; ^2^ Molecular Toxicology, Institute for Medical Research and Occupational Health, Zagreb, Croatia

**Keywords:** Pals1, Mpp5, solute carrier (SLC) family, SLC, kidney, nephron, SGLT2

## Abstract

Pals1 is part of the evolutionary conserved Crumbs polarity complex and plays a key role in two processes, the formation of apicobasal polarity and the establishment of cell-cell contacts. In the human kidney, up to 1.5 million nephrons control blood filtration, as well as resorption and recycling of inorganic and organic ions, sugars, amino acids, peptides, vitamins, water and further metabolites of endogenous and exogenous origin. All nephron segments consist of polarized cells and express high levels of Pals1. Mice that are functionally haploid for Pals1 develop a lethal phenotype, accompanied by heavy proteinuria and the formation of renal cysts. However, on a cellular level, it is still unclear if reduced cell polarization, incomplete cell-cell contact formation, or an altered Pals1-dependent gene expression accounts for the renal phenotype. To address this, we analyzed the transcriptomes of Pals1-haploinsufficient kidneys and the littermate controls by gene set enrichment analysis. Our data elucidated a direct correlation between TGFβ pathway activation and the downregulation of more than 100 members of the solute carrier (SLC) gene family. Surprisingly, Pals1-depleted nephrons keep the SLC’s segment-specific expression and subcellular distribution, demonstrating that the phenotype is not mainly due to dysfunctional apicobasal cell polarization of renal epithelia. Our data may provide first hints that SLCs may act as modulating factors for renal cyst formation.

## Introduction

The main tasks of mammalian kidneys are the filtration of the blood to excrete noxious substances, the resorption and recycling of nutrients, the control of salt and ion homeostasis and finally, the concentration of the primary ultra-filtrate into the secreted urine. These functions are carried out by up to 1.5 million nephrons, which are physiological subunits of mammalian kidneys. Each nephron contains a filtration unit with the glomerular filtration barrier and a tubular system composed of various segments, including the proximal tubule, the loop of Henle, and the distal tubule (Kobayashi et al., 2008; Kopan et al., 2014).

Nephron development begins with the renal vesicle stage when condensed pre-tubular mesenchymal cell aggregates start to form renal vesicles around the tip of the ureteric bud (Kobayashi et al., 2008; Kopan et al., 2014). The physiological function of various nephron segments requires the polarization of renal epithelial cells, ensuring the asymmetric distribution of lipids and proteins of the plasma membrane. This is of particular importance, as the tubular part of the nephron controls the specific recycling and reuptake of nutrients, salt homeostasis and water reabsorption.

During the last two last decades studies done in the fly *Drosophila melanogaster*, the zebrafish *Danio rerio* and particularly in mammalian cell lines elucidated the crucial role of the Crumbs protein complex for apicobasal cell polarization and junction formation (Pieczynski and Margolis, 2011; Rodriguez-Boulan and Macara, 2014). This complex consists of four proteins: the name-giving type I transmembrane protein Crumbs and three intracellular adapter proteins, called Lin7c (lin-7 homolog C), Pals1 (protein associated with Lin7, 1) and Patj (Pals1-associated tight junction protein) (Pieczynski and Margolis, 2011; Martin et al., 2021). In mammals exist three Crumbs isoforms: Crb1, Crb2, and two splice variants of the Crb3 isoform (Crb3a and Crb3b) (Pieczynski and Margolis, 2011; Martin et al., 2021). In the kidney Crb2, Crb3 and polarity components Pals1, Patj and Lin7c are expressed (Kamberov et al., 2000; Makarova et al., 2003; Olsen et al., 2007; Duning et al., 2008; Yin et al., 2014; Hochapfel et al., 2017). Crb3 isoforms are the main isoforms of the renal tubules (especially Crb3a), whereas parietal cells and podocytes of renal glomeruli express high levels of Crb2 (Hamano et al., 2018; Martin et al., 2021; Möller-Kerutt et al., 2021). Of note, Pals1 is the only protein that binds to all core components of the Crumbs complex (Kamberov et al., 2000; Roh et al., 2002; Roh et al., 2003).

In addition, knockdown studies in cell culture revealed an essential role of Pals1 in the formation of tight and adherence junctions, indicating that cell junction assembly and cell polarization are closely connected biological processes (Straight et al., 2004; Wang et al., 2007; Tan et al., 2020).

The polarization starts at the renal vesicle stage, but it is poorly understood how cell polarity is linked to a nephron-segment specific expression of genes and how dysfunction of these processes might be linked to renal diseases. We hypothesize, first, that key components of the Crumbs complex could be involved in the control of the spatial and temporal orchestration of these processes, and second, that disturbances in the coordination of these functions may provide novel insights into the pathomechanisms for renal disorders, particularly for cystic renal diseases. Recently, we addressed this aspect *in vivo* by using the *Six2*-Cre driver line in combination with conditional Pals1 knockout mice (*Pals1* ^flox/flox^) (Kobayashi et al., 2008; Kim et al., 2010; Weide et al., 2017). The homeodomain transcriptional regulator Six2 plays a key role during nephrogenesis and is expressed at the pre-tubular or cap mesenchymal stage before renal vesicles are formed (Oliver et al., 1995; Kobayashi et al., 2008). Therefore, mice that express Cre-recombinase under the control of the *Six2* promotor allow a specific gene targeting of all nephron epithelia, except for the collecting duct (Kobayashi et al., 2008).

Remarkably, already the deletion of one Pals1 allele was sufficient to cause lethality within the first 6–8 weeks after birth. Pals1-deficient-mice (Pals1^flox/wt^ × Six2-Cre) showed severe proteinuria, due to damages of the renal filtration barrier, as well as the formation of numerous cysts in different nephron segments (Weide et al., 2017). The haploinsufficiency of Pals1 in this mouse model argues for a gene dosage effect. This in turn suggests that a reduced Pals1 expression rather than a complete loss of Pals1-associated functions account for the fully penetrant phenotype. However, on cellular level, it is still unclear if the phenotype in Pals1-haplodeficient kidneys is mainly due to *defective* cell polarization, or *defective* cell-cell contact formation, or a combination of both. Moreover, increasing evidence suggests Pals1 (or the Crumbs complex) as a signaling hub for different downstream signaling pathways. This indicates that altered gene expression caused by reduced Pals1 protein levels may contribute as an additional relevant factor for the onset and progression of the Pals1 phenotype.

This study focuses on this aspect. We analyzed the transcriptomes of Pals1-haploinsufficient mice and the littermate controls by gene set enrichment analysis (GSEA) to seek for further genes and pathways that may act as co- or aggravating factors for the phenotype.

Strikingly, our analyses elucidated a direct correlation between TGFβ pathway activation and the downregulation of a high number of transporters of the solute carrier (SLC) gene family. This superfamily includes up to 458 transport proteins that can be subdivided into more than 60 subfamilies, and include some physiologically and pharmacologically interesting members like the glucose transporter SGLT2/Slc5a2 (Drozdzik et al., 2021; Pizzagalli et al., 2021). SLCs act as ATP-independent, passive-facilitative transporters or secondary-active transporters and serve as “gatekeepers” for low molecular weight molecules including sugars, amino acids, oligopeptides, vitamins, nucleotides as well as organic and inorganic ions and drugs (Drozdzik et al., 2021; Pizzagalli et al., 2021). At the plasma membrane of renal nephron epithelia, SLCs orchestrate the recycling, resorption, and secretion of these substrates to produce excretable urine from the glomerular ultra-filtrate. Therefore, this study focusses on the link between reduced Pals1 levels in the nephron and its impact on expression and localization of these transporters.

## Material and Methods

Animals and experimental design: Animals in this work involving Pals1 conditional knockout and Six2-Cre transgenic mice have been described earlier (Kobayashi et al., 2008; Kim et al., 2010; Weide et al., 2017). Animals were housed under standard specific pathogen–free conditions with free access to tap water and standard animal chow in accordance with all guidelines and regulations. All animal studies were performed in compliance with the ARRIVE guidelines and conducted in accordance with the *Guide for the Care and Use of Laboratory Animals* of the National Institute of Health. The studies were approved by the German regional authorities (Approval Number: Az: 84–02.04.2014 A405; LANUV).

### Gene Set Enrichment Analyses and Evaluation of Data

The platform *GO*rilla (from Gene Ontology enrichment analysis and visualization tool, Eden et al., 2007; Eden et al., 2009) was used for GSEA studies based on an earlier described DNA microarray-based transcriptome analysis of Pals1-deficient kidneys (Weide et al., 2017). *GO*rilla is a web-based tool with two application modes. It can either be used for the discovery of enriched gene ontology (GO) terms by comparing a target set of genes against a background set using the well-established hypergeometric model or it discovers enriched GO terms of a list of ranked genes by using mHG statistics, a method unique for this platform. Advantages of the platform *GO*rilla in comparison to other GO enrichment analysis tools are the enabling of a flexible threshold combined with an exact *p*-value for the detected event, the graphical representation of the data, and the combination of highly interactive settings with a processing time of only a few seconds per analysis. In this study, a target set of genes (genes from the DNA microarray-based transcriptome analysis above a fold change threshold of ±1.5) was tested against the background set of genes (all candidates of the micro array independent on the value). The *p*-value threshold was set to 10^−3^ and enrichment analysis was performed for the three ontologies biological process, molecular function, and cellular component.

For better visualization of the results after using *GO*rilla to analyze the GO terms of the array the platform Revi*GO* (from: *re*duce and *vi*sualize *G*ene *O*ntology) was used (Supek et al., 2011). It forms GO term clusters and shows only representatives of each cluster to make interpretation simpler and to reduce redundancy. In this process, Revi*GO* prioritizes statistically significant and more enriched terms. The results can be visualized using different graphs: Scatterplot, “interactive graph”, TreeMap, TagClouds. In this study, the scatterplot was chosen for the best visualization. It shows the cluster representatives by using a two-dimensional space whereas semantically similar GO terms are shown graphically closer. The color of the circles indicates the *p*-value and the size of the circles give some indication of the frequency of the GO term in this group.

### Quantitative Real-Time RT-PCR Analysis and Evaluation

The quantitative real time RT-PCR was done as described earlier (Weide et al., 2017; Möller-Kerutt et al., 2021). In brief, total RNA from mouse tissues was isolated using the GenElute™ Mammalian Total RNA Miniprep Kit (Sigma-Aldrich), according to the manufacturer’s instructions. Aliquots of total RNA (1–2 µg) were converted into cDNA using the SuperScript III Reverse Transcription Kit (Invitrogen, Darmstadt, Germany) according to the manufacturer’s instructions. For quantitative real-time RT-PCR the SYBR Green PCR Master Mix (Life Technologies) in combination with the Biorad CFX384 Touch (Bio-Rad Laboratories GmbH, Munich), and Bio-Rad CFX Manager v3.0 software was used. Relative expression levels of genes of interest were calculated as (∆C_T_) values normalized to the GAPDH control. Differences between expressions were calculated as ∆∆C_T_ value (fold change). (Livak and Schmittgen, 2001). The sequences of the primers used are listed in the Supplemental Material 1.

### SDS-PAGE and Western Blot Analyses

To perform Western Blot Analyses the kidneys were immersed in lysis buffer (5 ml Triton × 100, 10 ml Tris-HCl, 1 M pH 7.4, 25 mM NaCl, 50 mM NaF, 15 mM Na_4_P_2_O_7_) with additional protease and phosphatase inhibitors. The kidneys were homogenized with a tissue grinder and then pushed 10 times through a 20 gauge needle. The lysates were centrifuged at 13,000 g for 10 min. The supernatants were mixed with 2× Laemmli buffer and incubated at 95°C for 5 min. The following steps were performed as described earlier (Weide et al., 2017; Möller-Kerutt et al., 2021). In brief, equal volumes of lysates were fractioned on 10% SDS-PAGE gels at 150–200 V. In addition, a molecular size marker was loaded to identify the protein size. After that, the proteins were transferred onto a PVDF membrane using the semi-dry method. Next, the PVDF membrane was pre-incubated in blocking solution (5% BSA in TBS-T) for 1 h to avoid unspecific binding. The membrane was then incubated with the primary antibody overnight at 4°C. We used monoclonal antibodies (mAB) from Santa Cruz Biotechnology against Slc22a13 (sc-390931; 1:1:500). Polyclonal antibodies (pAB) were used against Slc34a3 (Aviva Systems Biology Corporation, ARP32173 P050, 1:500), Slc5a2 (Novus Biologicals, NBP1-92384, 1:500) and Slc16a14 (Sigma-Aldrich, HPA040518, 1:500). As loading control we used mAb against *α*-Actinin-4 (Enzo Life Science, 1:1,000), or a pAb against Actin (Sigma-Aldrich, 1:1,000). After 24 h the membrane was washed three times in TBS-T. For detection we applied secondary antibodies from Jackson Immunoresearch Laboratories coupled to horseradish peroxidase against mouse (HRP-α-mouse IgG; Jackson Immunoresearch Laboratories, 1:2,000), or rabbit (HRP-α-rabbit IgG, 1:2,000). The membrane was incubated for 1 h with the secondary antibody and washed again 3 times in TBS-T. In the last step, the targeted antigen was visualized using Lumi-Light (Roche) according to the manufacturer’s instructions.

### Immunohistologic Analyses Using Cryo-Sections

Kidney frozen sections (4–5 μm) were prepared in a cryostat and mounted on slides as detailed previously (Breljak et al., 2016; Weide et al., 2017). Immunohistologic analyses using cryo-sections for staining samples with Slc5a2 (Novus working dilution, 1:50, in 1% BSA antibody solution) and *Lotus tetragonolobus* Lectin (LTL) coupled to Fluorescein (Vector Laboratories, 1:200) were performed as described earlier (Weide et al., 2017). Commercial mAb for the Na/K-ATPase α1-subunit (sc-48345; 1:100) and *β*-actin (sc-47778; 1:20) were purchased from Santa Cruz Biotechnology, Inc (Santa Cruz, CA, United States) and their use was described previously (Breljak et al., 2016). Commercial secondary antibodies CY3-labeled goat anti-rabbit IgG (GAR-CY3; 1:800) and fluorescein isothiocyanate-labeled donkey anti-mouse IgG (DAM-FITC, 1:50) were purchased from Jackson Immuno Research Laboratories Inc. (West Grove, PA, United States).

### Statistical Analyses

The evaluation was done using GraphPad software. All data show SD of at least three independent experiments and were analyzed using unpaired Mann-Whitney *U* test.*p < 0.05; **p < 0.01; ***p < 0.001.

## Results

### GSEA of Pals1-Deficient Kidneys

**FIGURE 1:**
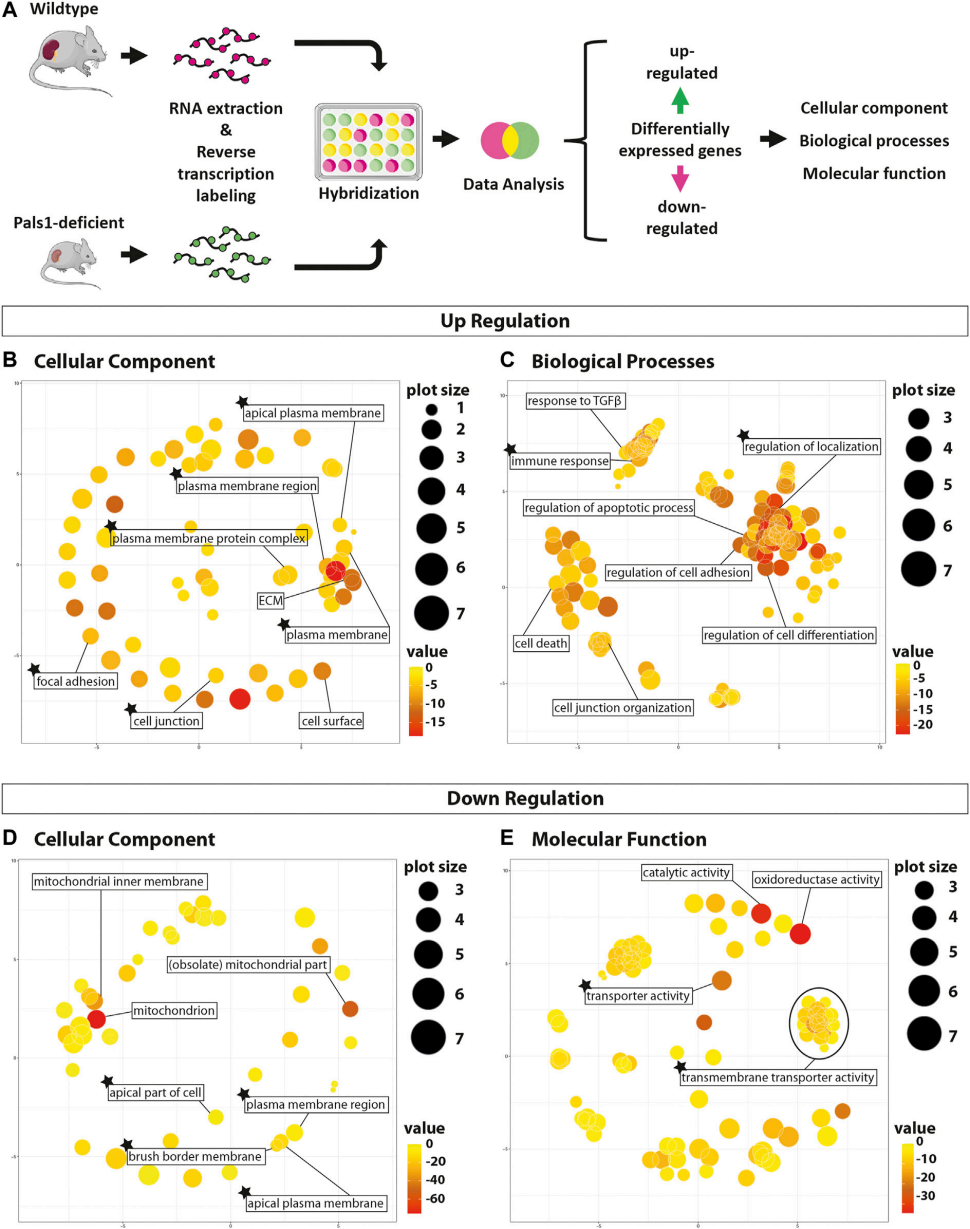
Gene set enrichment analyses of Pals1-depleted nephron epithelia*.* Gene set enrichment analyses (GSEA) of differentially regulated genes in Pals1-deficient kidneys. **(A)** Workflow of the study: After isolation of mRNA from Pals1-deficient kidneys and their littermate controls mRNA was reverse transcribed, labeled and subsequently analyzed by a gene set enrichment analyses for up- and downregulated differentially expressed genes (DEGs) using *GO*rilla and Revi*GO in silico* tools. **(B/C)** Revi*GO* images demonstrating the enrichment of GO terms of the categories *cellular component* (**B**; for details see Supplementary Material 2) and biological processes (**C**; Supplementary Material 3). **(D/E**) Revi*GO* schemes: GO terms of categories cellular components (**D;** Supplementary Material 5) and molecular functions (**E**; Supplementary Material 7) that were matched by downregulated DEGs of Pals1-deficient kidneys. The heat map indicates the *p*-value. The plot size indicates the number of regulated genes that match the different GO terms. The asterisks marks GO subsets including transporters of the SLC family.

The comparison between heterozygote *Pals1* ^*flox/wt*^ *Six2-*positive mice and their littermate controls (we were unable to establish mice lacking both alleles) resulted in more than 1,600 differentially expressed genes (DEGs) (Weide et al., 2017). In this study, we re-evaluated Pals1-dependent gene expression by taking advantage of the *GO*rilla tool (Eden et al., 2007; Eden et al., 2009) and compared the Pals1-dependent up- or downregulated DEGs with the *a priori* defined gene ontology (GO) categories *cellular component*, *biological process,* and *molecular function* (Ashburner et al., 2000; Carbon et al., 2021) (Figure 1A).

Upregulated DEGs of Pals1-deficient kidneys are linked to 68 GO terms of the *cellular component* (GO-CC, Supplementary Material 2), to 649 GO terms of *biological process* (GO-PB, Supplementary Material 3) and to 46 GO subsets to the *molecular function* (GO-MF, Supplementary Material 4) category. Strikingly, the GO-CC subsets are for example connected to the cell surface (GO:0009986), the (apical) plasma membrane (GO:0005886, GO:0016324, GO:0005903), cell-cell junctions (GO:0030054, GO:0005912) or the extracellular matrix (GO:0031012), which is necessary for cell adhesion (GO:0005925). This fits to the known role of Pals1 as a component of the apical polarity complex and its proposed function in cell-junction formation (Figure 1B; Supplementary Material 2).

Identified GO subsets of the GO-BP (Figure 1C; Supplementary Material 3) and GO-MF categories (Supplementary Materials 4 and 8) include the subsets TGFβ response (GO:0071559), TGFβ receptor (GO:0005160) and SMAD binding (GO:0046332, GO:0070412), and processes that have been indirectly linked to TGFβ signaling, such as regulation of development and differentiation (*e.g.,* GO:0050793, GO:0045595), cell migration and motility (*e.g.,* GO:0040012, GO:0030334, GO:0030335), the control of cell adhesion (e.g., GO:0030155), the regulation of programmed cell death (*e.g.,* GO:0010941, GO:0042981, GO:0008219), the control of inflammatory and immune responses (*e.g.,* GO:0006954, GO:0050776) as well as cell junction formation (*e.g.,* GO:0034329, GO:0034330, GO:1901888, GO:1903391, GO:0007043). These data are in line with a previous study, demonstrating that Pals1 deficiency in the kidney leads to an upregulation of renal injury marker genes and target genes of TGFβ and Hippo signaling pathways (Weide et al., 2017).

The downregulated DEGs were enriched in 62 GO terms of the GO-CC category (Figure 1D; Supplementary Material 5). Among them are subsets that are linked to mitochondria (*e.g.,* GO:0005739, GO:0044429, GO:0005743), the plasma membrane (GO:0098590, GO:0016323), in particular the apical membrane (GO:0016324, GO:0045177), the brush border (*e.g.,* GO:0031526, GO:0005903), and the slit diaphragm of podocytes (GO:0036056, GO:0036057). This suggests that the integrity of the brush border membrane of the proximal tubular epithelial cells and the slit diaphragm formed by glomerular podocytes depends on the expression of Pals1 (Figure 1D; Supplementary Material 5).

In the GO-BP category downregulated DEGs of Pals1-deficient kidneys could be linked to more than 250 GO terms, most of these subsets (87%; 223 out of 256; Supplementary Materials 6 and 8) are connected to GO terms addressing metabolism (106 GO terms), catabolism (26 terms) biosynthesis (45 terms) and the transport (40 terms, excluding GO terms addressing electron transport) of small molecules. The 119 GO terms of the GO-MF category showed an enrichment of GO subsets linked to the binding of small substrates (37 out of 119) and 28% (33 out of 119 GP terms) are connected to transmembrane transporter activities (Figure 1E; Supplementary Materials 7 and 8).

### The SLC Family Is Strongly Regulated in Pals1-Deficient Kidneys

Taking a view into the individual gene lists of the GO-MF and -BP categories, revealed that many of the enriched genes, particularly of downregulated DEGs, encode for members of the SLC gene family.

Indeed, Pals1 deficiency in the kidney resulted in significantly changed expression of one-third (120 out of 375) of the SLC members with 19 genes being up- and 101 genes downregulated (Supplementary Material 9). Upregulated SLC genes show a rather moderate increase of expression levels compared to the Cre-positive wildtype (>1.5 to <5 fold upregulated; see Supplementary Material 9). In contrast, of the 101 genes, 89 were moderately (>1.5 to <5) and 12 strongly (>5fold) downregulated (see Table 1 and Supplementary Material 9).

**TABLE 1:** *SLC genes are strongly downregulated in Pals1 haploinsufficient kidneys.* More than 100 genes of the SLC superfamily (Pizzagalli et al., 2021) are downregulated in Pals1-haploinsufficient kidneys (see Supplementary Material 9; Weide et al., 2017). The table shows ten SLC genes *i*) that are known to be highly expressed in the kidney, *ii*) functionally and phylogenetically conserved in mammalian species (mouse, dog, human) and *iii*) among the most regulated genes identified in the transcriptome of Pals1-deficient kidneys.

*Gene*	Alias	Function	Nephron localization	Fold change	References
*Slc5a2*	SGLT2	Sodium glucose cotransporter	Proximal tubules at the apical brush border (BBM) membrane	**−5.6**	Vallon et al. (2011), Wright et al. (2011), Sabolić et al. (2012), and Vrhovac et al. (2015)
*Slc10a2*	ASBT, IABT, ISBT, NTCP2	Sodium bile salt cotransport	Proximal tubules at the apical brush border (BBM) membrane	**−5.3**	Hagenbuch and Dawson (2004)
*Slc16a4*	MCT4,MCT5, MOT5	Monocarboxylate transporter	Unknown (basolateral in MDCK cells)	**−9.1**	Deora et al. (2005) and Halestrap (2013)
*Slc16a14*	MCT14, MOT14		Human: Unknown Mouse: TALH	**−5.4**	Halestrap (2013) and Knopfel et al. (2017)
*Slc22a2*	OCT2	Organic cation/anion/zwitterion transporter family	Proximal tubules at the basolateral membrane (BLM)	**−2.9**	Holle et al. (2011), Koepsell (2013), and Schulze et al. (2017)
*Slc22a7*	OAT2, NLT		Proximal tubules at BLM	**−13.8**	Nigam et al. (2015) and Breljak et al. (2016)
*Slc22a8*	OAT3		Proximal tubules at the BLM	**−4.4**	Nigam et al. (2015) and Breljak et al. (2016)
*SLC22a13*	OCTL1, OCTL3, OAT10, ORCTL3		BLM of type A intercalated cells (rat)	**−3.7**	Schulz et al. (2014)
*SLC34a3*	NPT2c	Type II sodium-phosphate cotransporter	Proximal tubules at the apical BBM membrane	**−11.2**	Dasgupta et al. (2014) and Levi et al. (2019)
*SLC39a5*	ZIP5, LZT-Hs7	Metal (Zinc) ion transporter	unknown	**−3.9**	Wang et al. (2004) and Jeong and Eide (2013))

Most affected are SLC subfamilies that transport sugar in particular glucose (SLC2 and SLC5 subfamilies), the sodium- and chloride-dependent neurotransmitter transporter family (SLC6 group), the amino acids transporters (SLC7 group), monocarboxylate transporters (SLC16 group), and organic cat-, an-, zwitterions transporters (SLC22 group). Differentially regulated are also all three genes encoding type II sodium-phosphate cotransporters of the SLC34 family. Moreover, the expression of numerous members of the mitochondrial carrier family (SLC25 subfamily), which control for example the transport of amino acids, carboxylic acids, fatty acids, inorganic ions, or nucleotides across the mitochondrial inner membrane (Kunji et al., 2020), was changed in Pals1-deficient kidneys (Supplementary Material 9).

In the following step, we focused on genes that are known to be highly (or almost exclusively) expressed in the kidney, that are phylogenetically and functionally conserved in mammalian species (mouse, dog, human), and that are among the most regulated genes identified in the GSEA approach. Applying these criteria resulted in ten genes (Table 1), including the glucose transporter Slc5a2 (Vallon et al., 2011; Wright et al., 2011), the sodium bile salt co-transporter Slc10a2 (Hagenbuch and Dawson, 2004), the monocarboxylate transporters Slc16a4 and Slc16a14 (Halestrap, 2013; Knopfel et al., 2017), organic cation/anion/zwitterion transporters of the SLC22 group, Slc22a2, Slc22a7, Slc22a8, and (Koepsell, 2013; Nigam et al., 2015; Breljak et al., 2016) the Slc34a3 sodium-phosphate cotransporter (Levi et al., 2019), and the putative zinc-transporter Slc39a5 (Jeong and Eide, 2013).

**FIGURE 2:**
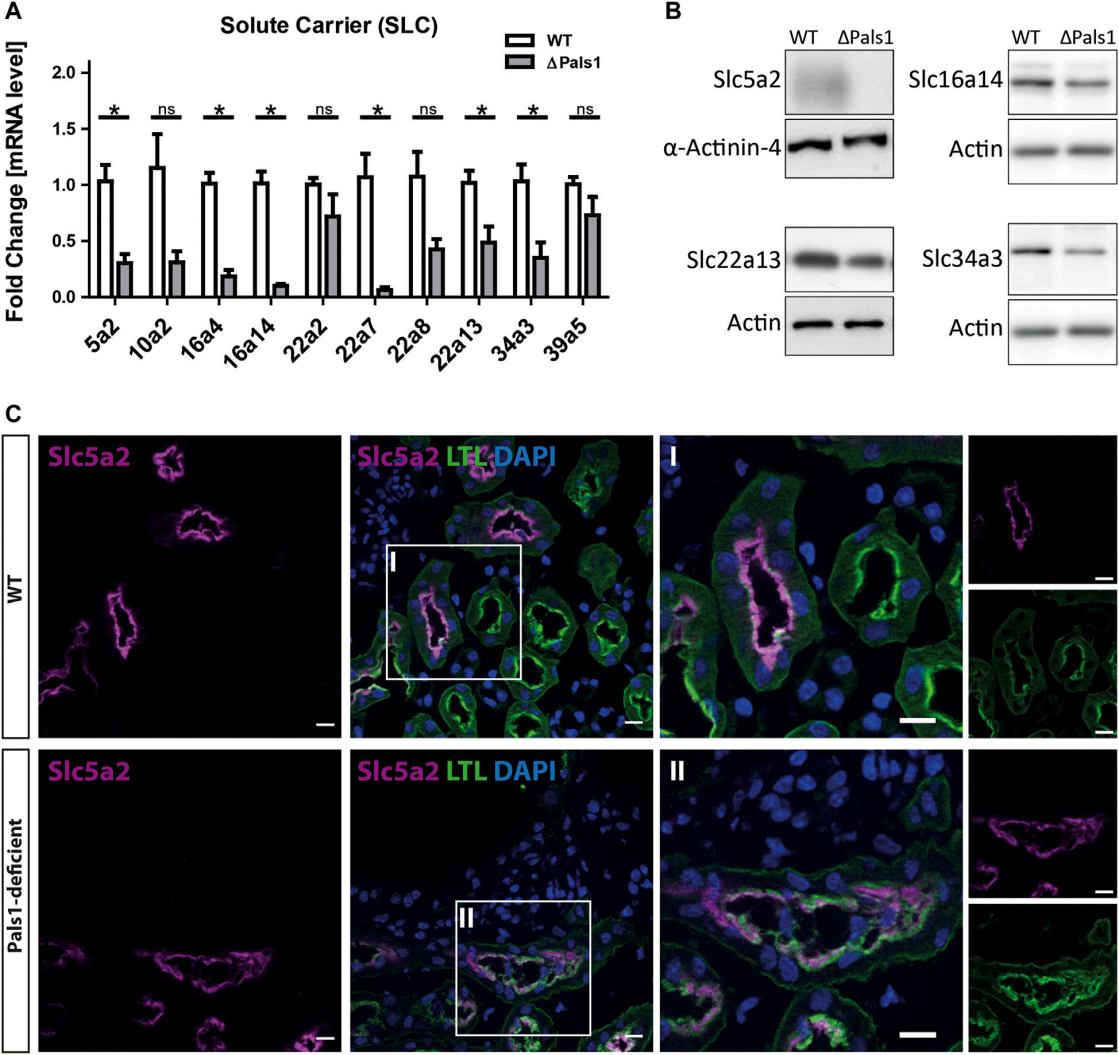
Pals1-dependent expression of abundant members of the solute carrier family members in renal nephrons. **(A)** Quantitative real-time RT PCR analysis of mRNA levels derived from Pals1-deficient kidneys (∆Pals1) and their wildtype (WT) littermate controls. In Pals1-deficient kidneys, six SLC genes summarized in Table 1 show a reduced mRNA expression in Pals1-deficient kidneys. **(B)** Western blotting confirmed downregulation on protein level for Slc5a2, Slc16a14, Slc22a13 and Slc43a3. α-Actinin-4 and Actin served as loading controls. (C) Immunohistologic analysis from kidney sections derived from WT and Pals1-deficient mice. The glucose transporter Slc5a2 (magenta) co-localizes with the *Lotus tetragonolobus* Lectin coupled to fluorescein (LTL, green). The lectin LTL is a marker for proximal tubules in mammalian kidneys. DAPI (blue) labels the nuclei of cells. Although downregulated, Slc5a2 glucose transporters localize at the brush border membrane of proximal tubules in Pals1-deficient mice. Bar = 10 μm.

Next, we prepared mRNA from mice with Pals1-deficient kidneys (Supplementary Material 10) and their littermate controls to confirm the downregulation of these genes at mRNA level by quantitative real-time RT-PCR analysis. As an internal setup control, we included genes corresponding to the TGF pathway (Serpine1 or Pai-1) and Hippo-TGF pathway (*Ctgf* and *Cyr61*), as well as known markers of renal injury (Lcn2/Ngal) and inflammation (Ccl2) in the quantitative real-time RT-PCR analysis. In Pals1-deficient kidneys, Lcn2 and Ccl2 as well as Serpine1, Ctgf and Cyr61 genes were strongly upregulated (Supplementary Material 8). In contrast, six of the ten selected gene of the SLC family (Slc5a2, Slc16a4, Slc16a14, Slc22a7, Slc22a13 and Slc34a3) showed a significant downregulation at mRNA level following Pals1 depletion, whereas the other tested SLC genes (Slc10a2, Slc22a2, Slc22a8 and Slc39a5) showed a trend toward downregulation, but no significance (Figure 2A).

To further address how an altered mRNA expression may cause effects on the protein expression, we performed Western blot analysis with antibodies against Slc5a2, Slc16a14, Slc22a13 and Slc34a3 (analyses of other SLCs were not performed due to a lack of specific antibodies). These Western blot data show reduced levels for these SLC proteins in Pals1-deficient kidneys (Figure 2B.), thus confirming the quantitative real-time RT-PCR data of the mRNA level as shown in Figure 2A.

### Nephron Segment-specific Expression of SLC Proteins (Slc5a2, Slc22a7 and Slc22a8) is Maintained in Pals1-Deficient Kidneys

**FIGURE 3:**
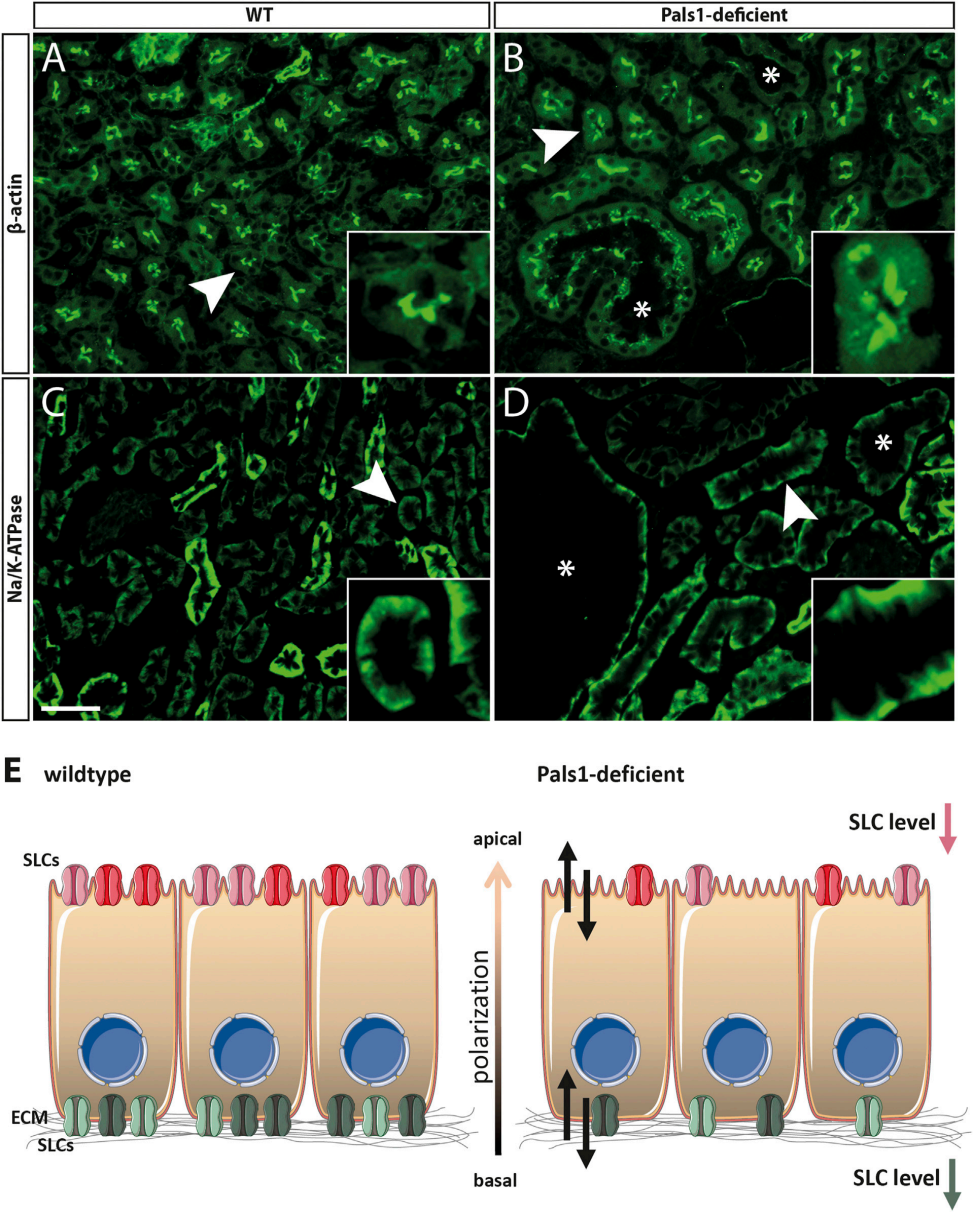
*Pals1 as a putative regulator of SLC expression in the kidney*. **(A–D)**: Immunofluorescence analyses of the kidney cortex of wildtype (A/C) and Pals1-deficient mice (B/D): The anti β-Actin antibody strongly stained the brush border membrane of proximal tubules, in both, wildtype **(A)** and Pals1-deficient mice **(B)**. **(C,D)** Immunofluorescence using an antibody against Na/K-ATPase stained the basolateral membrane of renal proximal tubules in wildtype **(C)** and Pals1-deficient kidneys **(D)**. Asterisks: cyst; Bar = 20 µm **(E)** Scheme: The physiological function of SLCs expressed in the nephron is the reabsorption and secretion of multitude substrates including ions, sugars, amino acids, peptides, vitamins, and various metabolites of endogenous and exogenous origin (see Introduction). The reduced mRNA/protein expression of nephron-specific SLCs in Pals1-deficient epithelia could be linked with imbalanced homeostasis of the nephrons’ intraluminal fluid composition (double arrows). This in turn might modulate the Pals1-associated phenotype. (Parts of the image were created using *smart. servier.com*).

Immunohistochemical (IHC) examinations were performed by using antibodies against Slc5a2 (Figure 2C), Slc22a7 and Slc22a8 and markers of the apical (β-actin) and the basolateral (Na^+^/K^+^-ATPase) membranes of renal epithelia (Figures 3A–D; Supplementary Material 11).

The fluorescence intensity of Slc5a2 was reduced in Pals1-deficient kidneys (Figure 2C; Supplementary Material 11). However, as shown in Figure 2C, both sections of wildtype (Cre-negative) as well as Pals1-deficient (Cre-positive) kidneys showed a co-localization of the glucose transporter Slc5a2 with *Lotus tetragonolobus* lectin (LTL), which predominantly localizes at the apical membrane domains of proximal tubule epithelial cells in mouse and human kidneys (Schulte and Spicer, 1983).

Furthermore, IHC analysis clearly showed that Slc5a2 (Figure 2C; Supplementary Material 11) and SLC22a7 (SM11c-d) retained their original localization pattern at the apical side of epithelial cells in the proximal tubules of both Pals1-deficient and wildtype kidneys. In addition, IHC analysis showed that the Slc22a8 preserved its basolateral membrane localization in the proximal tubules of both wildtype and Pals1-deficient kidney (SM11e-f). The apical marker *β*-actin and the basolateral marker Na/K-ATPase keep their polarized distribution in the renal cortex segments showing the same localization pattern at the plasma membrane in Pals1-deficient and wildtype epithelia (Figures 3A–D, Supplementary Material 11).

## Discussion

Reduced Pals1 levels in mouse experimental model cause more than 1,600 DEGs. Thus, Pals1-dependent gene expression patterns most likely not only include the strong up- or downregulation of marker genes, but also moderate changes of entire pathway-specific gene clusters. To address this, we tested to what extent up- and downregulated genes in the kidneys of Pals1-deficient mice are enriched in given *a priori* GO terms of the GO categories GO-CC, -BP, and MF (Ashburner et al., 2000; Carbon et al., 2021).

Particularly, upregulated DEGs matched GO terms of the *cellular component GO category* that are connected to the apical cell surface (*e.g.* apical plasma membrane, brush border, cell junction formation, and cell adhesion, see Figure 1A; Supplementary Material 2). Indeed, this emphasizes the known role of Pals1 as a regulator of apicobasal cell polarization and its proposed function in cell-cell contact establishment (Hurd et al., 2003; Roh et al., 2003; Straight et al., 2004; Wang et al., 2007; Tan et al., 2020). The significant upregulation of genes of these GO terms most probably reflects the attempt of cells to balance too low levels of Pals1 in the renal epithelia.

Numerous studies showed that tubular injury of nephron epithelia, including diabetic nephropathies, correlates with the expression of cytokines, apoptosis and de-differentiation processes (Edeling et al., 2016). Thus, enriched GO terms linked to cell locomotion and migration, responses of the immune system, and programmed cell death support this point of view and suggest that these processes most likely act as aggravating factors for the dramatic Pals1 phenotype.

Remarkably, the GO-MF category shows a direct correlation between *downregulated* Pals1 and *upregulation* of TGFβ-linked GO terms (*e.g.* TGF receptor-, SMAD-, R-SMAD-binding; see ST3). This data confirms earlier concepts in which TGFβ/SMAD signaling is activated in cells that undergo epithelial mesenchymal transition (EMT, see Varelas et al., 2010 and Weide et al., 2017) suggesting that Pals1 expression levels act as an upstream signaling hub for TGFβ and its downstream pathways and target genes (like *Serpine1*).

The downregulated DEGs show a strong enrichment of GO terms that are linked to physiological processes like metabolism, catabolism, or transport activities across the plasma membranes of cells. Interestingly, the GSEA revealed a conspicuous downregulation of more than 100 members of the SLC family in Pals1-deficient renal epithelia, with some of them (*e.g.* Slc5a2, Slc10a2, Slc16a4, Slc16a14, Slc22a7, or Slc34a32, see Table 1) being among the 50 most strongly downregulated DEGs. However, whereas Pals1 is expressed in all parts of the renal nephrons, most SLC family genes show a nephron segment-specific expression pattern (Table 1, Supplementary Material 11).

This argues for mechanisms in which members of the Crumbs complex or Pals1-associated cellular processes act as upstream regulators for SLC expression and not *vice versa*. Pals1 is a junction-associated protein and does not shuttle to the nucleus and is therefore unable to directly change gene expression as a transcription factor. Thus, details about how Pals1 levels are linked to the gene expression of SLC family members (at least of Slc5a2, 16a14, 22a13 and 34a3) will require further in-depth analyses.

So far, analyzed SLCs keep their nephron-segment specific expression and distribution in Pals1-depleted kidneys, even in cyst lining epithelia (Figure 2C, Figures, 3A–D, Supplementary Material 11). This supports the assumption that remaining Pals1 levels in nephron epithelia maintain an overall cell polarization. In previous concepts, the Crumbs complex was identified as cell density sensors for epithelial tissues, indicating that Crumbs complex components could be part of feedback loops in which reduced cell-cell contact formation may trigger an increased TGFβ signaling (Varelas et al., 2010; Weide et al., 2017). However, it remains to be shown how such mechanisms might be linked to an altered gene expression of SLC members (Figure 3E).

The loss of Pals1 or its binding partners (Crb3, Lin7c, or Taz) results in the dilation of tubules and the formation of cysts (Hossain et al., 2007; Olsen et al., 2007; Tian et al., 2007; Makita et al., 2008; Reginensi et al., 2013; Whiteman et al., 2014; Weide et al., 2017). This raises the question, how far an altered expression of the SLC family (or a single SLC member) is involved in the formation or enlargement of renal cysts. Thus, in case that transport activities of a group, or a single SLC family member significantly aggravate cyst formation, downregulation of SLCs in Pals1-depleted renal epithelia could be part of a protective “rescue” effect, preventing further (or faster) progression of the phenotype. *Vice versa*, the maintenance of SLC’s expression and transport activities may cause attenuating effects on onset or progression of the cyst formation. In such a scenario, SLC downregulation rather should be interpreted as an aggravating factor for cyst formation.

So far, both, attenuating and aggravating effects of SLC expression on renal cystic diseases are poorly investigated, and addressing them will require elaborate animal models, for example ADPKD (autosomal dominant polycystic kidney disease) mouse models, treated with agonist or antagonist of individual SLCs. A further approach could be the breeding of ADPKD mouse models with mouse lines lacking or overexpressing single SLCs.

However, there are at least some hints supporting the assumption that SLCs could be directly or indirectly involved in cystic diseases. For example, downregulation of SLC family members has also been observed in two further animal models that develop renal cysts: a murine model for the renal cysts and diabetes (RCAD) syndrome (Niborski et al., 2021) and a mouse model linked to Birt–Hogg–Dubé (BHD) syndrome (Centini et al., 2018). In addition, *Hurd et al.* observed that mutations within the human *SLC41A1* gene (encoding an Mg^2+^ transporter) cause a Nephronophthisis-like phenotype, leading to numerous renal cysts (Hurd et al., 2013). Furthermore, the CFTR (cystic fibrosis transmembrane conductance regulator), the Ca^2+^-activated chloride channels TMEM16A (Anoctamin 1), but also the SLC member Slc12a2 have been identified as crucial aggravating co-factors for cyst enlargement in polycystic kidney disease (Magenheimer et al., 2006; Cabrita et al., 2020). This indicates that an imbalanced ion transport across epithelial cells, in this case a chloride transport, can be a relevant trigger for renal cyst growth. Moreover, modulation of the Slc5a2 activity by inhibitors (SGLT2 inhibitors), which are promising drugs in the treatment of diabetes, including diabetic nephropathy (Vallon, 2011; Perkovic et al., 2019; Zelniker et al., 2019), may also modulate cyst growth in polycystic kidney diseases (Kapoor et al., 2015; Rodriguez et al., 2015; Patel and Dahl, 2021).

As mentioned above, SLCs are “gatekeepers” and are the main factors for the reabsorption and secretion of various small molecular weight substrates such as ions, sugars, amino acids, peptides, vitamins, and further metabolites of endogenous and exogenous origin (Pizzagalli et al., 2021). Therefore, an imbalanced expression of SLC family members may be linked to altered transport activities as well as to changed compositions and osmolalities of the nephrons’ intra-tubular lumen fluid (Figure 3E). This could be of relevance in cystic but also other renal diseases (*e.g.,* acute kidney injury, see Vallon, 2016, and references therein).

SLC localization in the apical or basolateral parts of the plasma membrane may provide direct access for drugs. Thus, especially in case that downregulation of SLC members provides a renoprotective potential, SLC inhibitors could be interesting “druggable” targets for renal diseases.

## Data Availability

The original contributions presented in the study are included in the article/Supplemental Material, further inquiries can be directed to the corresponding author.
