# Supplementary material for: Impact of Pals1 on Expression and Localization of Transporters Belonging to the Solute Carrier Family
Source: Front Mol Biosci. 2022 Feb 16;9:792829. doi: 10.3389/fmolb.2022.792829 (PMC8888964; doi:10.3389/fmolb.2022.792829)
Supplement: Supplementary file 4 [file DataSheet1.pdf]

SUPPLEMENTAL MATERIAL

Impact of Pals1 on expression and localization of transporters belonging to the solute carrier family

by

Carmen Berghaus[1*], Ann-Christin Groh[1*], Davorka Breljak[2], Giuliano Ciarimboli[1], Ivan Sabolic[2], Hermann Pavenstädt[1] and Thomas Weide[1§]

[1)] University Hospital of Münster (UKM), Internal Medicine D (MedD), Albert-Schweitzer-Campus 1 Building A14, Münster, 48149, Germany; [2)] Molecular Toxicology, Institute for Medical Research and Occupational Health, Ksaverska cesta 2, 10000 Zagreb, Croatia

[*)] contributed equally; [§)] corresponding author

Table of content (supplemental material, SM)

SM1 Used primers for quantitative real time PCR

SM2 Matched *cellular components* GO subsets for upregulated DEGs

SM3 Matched *biological processes* GO subsets for upregulated DEGs

SM4 Matched *molecular function* GO subsets for upregulated DEGs

SM5 Matched *cellular components* GO subsets for downregulated DEGs

SM6 Matched *biological processes* GO subsets for downregulated DEGs

SM7 Matched *molecular function* GO subsets for downregulated DEGs

SM8 Data set: Transcriptome analysis

SM9 Solute carrier gene family expression in Pals1-deficient kidneys

SM10 Data set: Used Pals1-deficient kidney specimen

SM11 Data set: IHC analysis of SLC family members (Slc5a2, Slc22a7 and Slc22a8) and Na/K-ATPase

SM1 *Used primers for quantitative real time PCR*

Primers used for genotyping and quantitative real time PCR analyses. Primers are given in 5'-3' orientation.

Gen	Forward primer	Reverse Primer	Application
Pals1	TTTTTCCCACTTCTCATTACAGTG	GCCCTCCGTTTCCTCTTATC	genotyping
Cre	GCATTACCGTCGATGCAACGAGTGATGAG	GAGTGAACGAACCTGGTCGAAAT CAGTGCG	genotyping
Serpine1a	CCGATGGGCTCGAGTATGAC	AGCTGCTCTTGGTCGGAAAG	qPCR
Ctgf	GTGTGCACTGCCAAAGATGG	ATTTCCCAGGCAGCTTGACC	qPCR
Lcn2	GACTTCCGGAGCGATCAGTT	CTGTACCTGAGGATACCTGTGC	qPCR
Cyr61	CACTGAAGAGGCTTCCTGTCTT	GATCCGGGTCTCTTTCACCA	qPCR
Ccl2	AGCTGTAGTTTTTGTCACCAAGC	GTGCTGAAGACCTTAGGGCA	qPCR
Slc5a2	CTAACATCGCCTACCCACGG	GAATACCACCCAGAGCCTTCC	qPCR
Slc16a4	ACCTGCTTGCTCCTTTAGCC	TCCACCCATGAGTTACATCTGC	qPCR
Slc16a14	CACCTTGATCGTGGGACCTT	CCACTGCAGGTAGGTAAGCC	qPCR
Slc10a2	GGGGTATCTTCGTGGGCTTC	TGGTCATGCTAACACTGAGGTC	qPCR
Slc22a2	AGCCTACGAAATGGTCTGCC	GCCAACCACAGCAAATACGA	qPCR
Slc22a7	CCCTACTGTGCTCAGACAGAC	CACGTCCTGGATGGTCTCTG	qPCR
Slc22a8	CCCTACAGTCCTCAGGCAAAC	CTGGTAAGGGCCGATTGAGG	qPCR
Slc22a13	CCCACCATCATCAGGCAAACA	GTGCTGTTTTGTGGGACCCT	qPCR
Slc34a3	TCAGCGGCTACTTGGCTATC	GATGAGAGCAACCTGAACTGC	qPCR
Slc39a5	GGCATTAGGTGCTGCTTTCTC	GCATATCCACTAGGGCCACA	qPCR
Gapdh	TGGCCTTCCGTGTTCCTACC	GGTCCTCAGTGTAGCCCAAGATG	qPCR
Actin	ACTATTGGCAACGAGCGGTTC	TTACGGATGTCAACGTCACACTTC	qPCR
Pals1	TCAGGCACTTTTACTGGCCC	AACTGTGGCACCCAATGGAA	qPCR

SM2-SM7 *Description of excel sheets*

Only in case that the DEGs (from: differentially expressed genes) enrichment is above the value 10^3 terms are included of the list. Labeling of columns: ***GO Term***: GO term number; ***Description***: name of GO term; **FDR** (q-Value) is the false discovery rate; Enrichment (N, B, n, b); **N** - is the total number of genes; **B** - is the total number of genes associated with a specific GO term; **n** - is the number of genes in the top of the user's input list or in the target set when appropriate; **b** - is the number of genes in the intersection.

The enrichment is defined as the ratio between (b/n) / (B/N). Highlighted in yellow are the SLC genes that can be found in the different categories.

SM2 *Matched cellular components GO subsets for upregulated DEGs.*
This excel sheet includes gene ontologies (GO) terms of the category *cellular compon*ents that were matches by differentially upregulated genes in Pals1-deficient kidneys.

SM3 *Matched biological processes GO subsets for upregulated DEGs.*
This excel sheet includes gene ontologies (GO) terms of the category *biological processes* that were matches by differentially upregulated genes in Pals1-deficient kidneys.

SM4 *Matched molecular function GO subsets for upregulated DEGs.*
This excel sheet includes gene ontologies (GO) terms of the category *molecular function* that were matches by differentially upregulated genes in Pals1-deficient kidneys.

SM5 *Matched cellular components GO subsets for downregulated DEGs.*
This excel sheet includes gene ontologies (GO) terms of the category *cellular components* that were matches by differentially upregulated genes in Pals1-deficient kidneys.

SM6 *Matched biological processes GO subsets for downregulated DEGs.*
This excel sheet includes gene ontologies (GO) terms of the category *biological processes* that were matches by differentially upregulated genes in Pals1-deficient kidneys.

SM7 *Matched molecular function GO subsets for downregulated DEGs.*
This excel sheet includes gene ontologies (GO) terms of the category *molecular function* that were matches by differentially upregulated genes in Pals1-deficient kidneys.

SM8

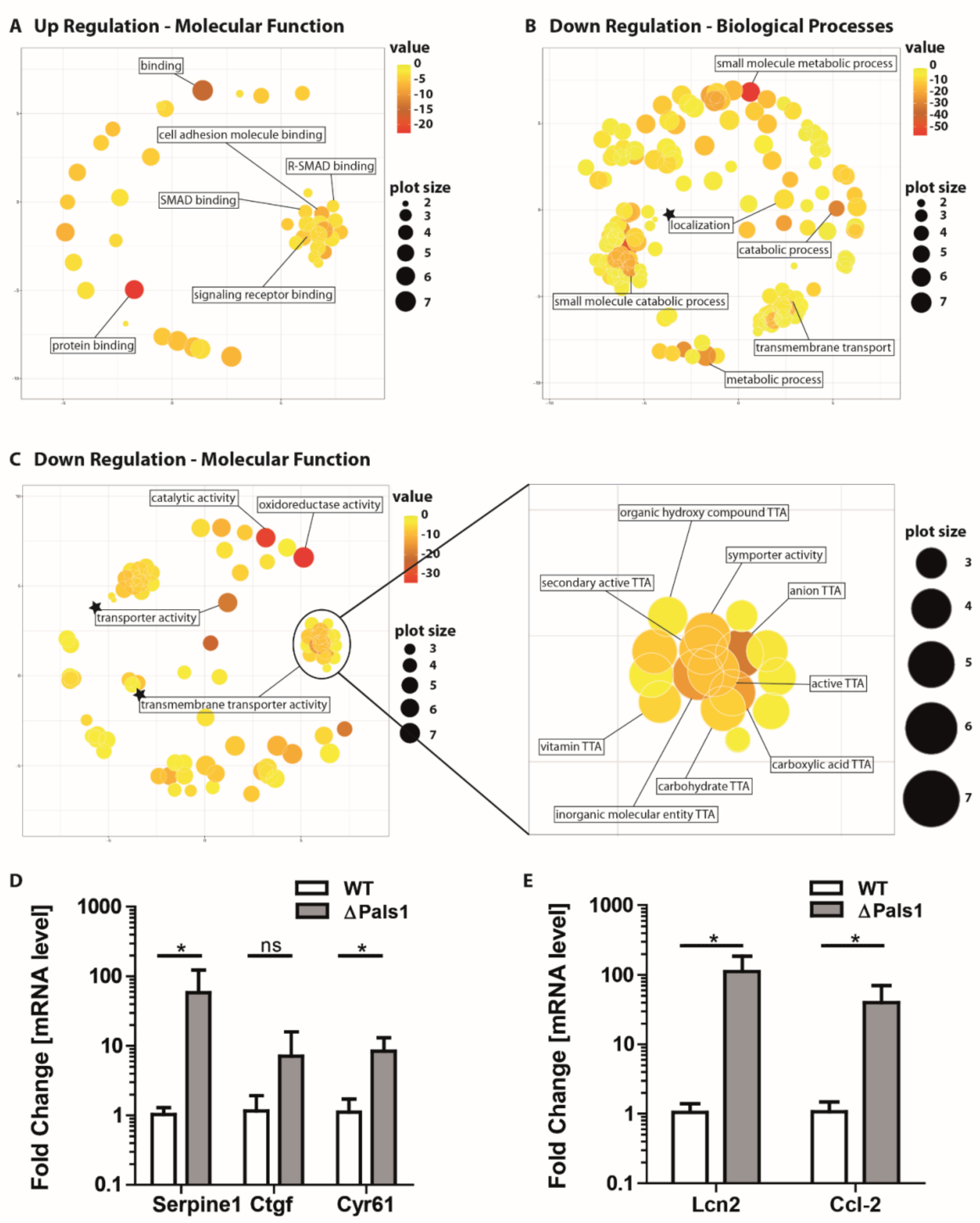

SM8: *Data set: transcriptome analysis*

Gene set enrichment analyses (GSEA) of differentially regulated genes in Pals1-deficient kidneys. **(A)** Revi*GO* images demonstrating the enrichment of GO terms of the categories *molecular function* for upregulated DEGs (for details: Table ST3). (**B**) Revi*GO* illustration of matched GO terms of downregulated DEGs in the category *biological processes* (for details: Table ST5). **(C)** GO terms of downregulated DEGs in the category molecular function showed a striking clustering of terms linked to transmembrane transporter activities (Zoom left site, for details ST6). The heat map indicates the p-value. The plot size indicates the number of regulated genes that match the different GO terms. The asterisks marks GO subsets including transporters of the SLC family. **(D/E)** Quantitative real-time RT-PCR analyses of mRNA levels derived from Pals1-deficient kidneys (ΔPals1) and their littermate controls (suppl. data set A). In Pals1-deficient kidneys target genes of the TGFβ (Serpine1) and Hippo-pathways marker genes Ctgf and Cyr61 (D) as well as renal injury markers Lcn2 and Ccl2 (E) are upregulated.

SM9 *Solute carrier gene family expression in Pals1-deficient kidneys*

This excel sheet summarizes all members of the SLC gene superfamily that are differentially expressed in the mouse kidneys following Pals1-silencing. The first column gives the SLC subfamily number, the second column the predicted role of the subfamily, and the third column the SLC members that are found in kidney transcriptomes of Pals1-haplodeficient mice and their littermate controls (Weide et al., 2017). The fourth column gives the fold change values of Pals1-deficient (Cre+) mice versus their littermate controls (Cre-). In red are fold change values marked that are more than 1.5fold, but below 5 (> 1.5x < 5x) regulated. Dark red values indicate fold changes above 5 (>5x). Upregulated SCLs are labeled in green. (There were no values above 5x).

SM10

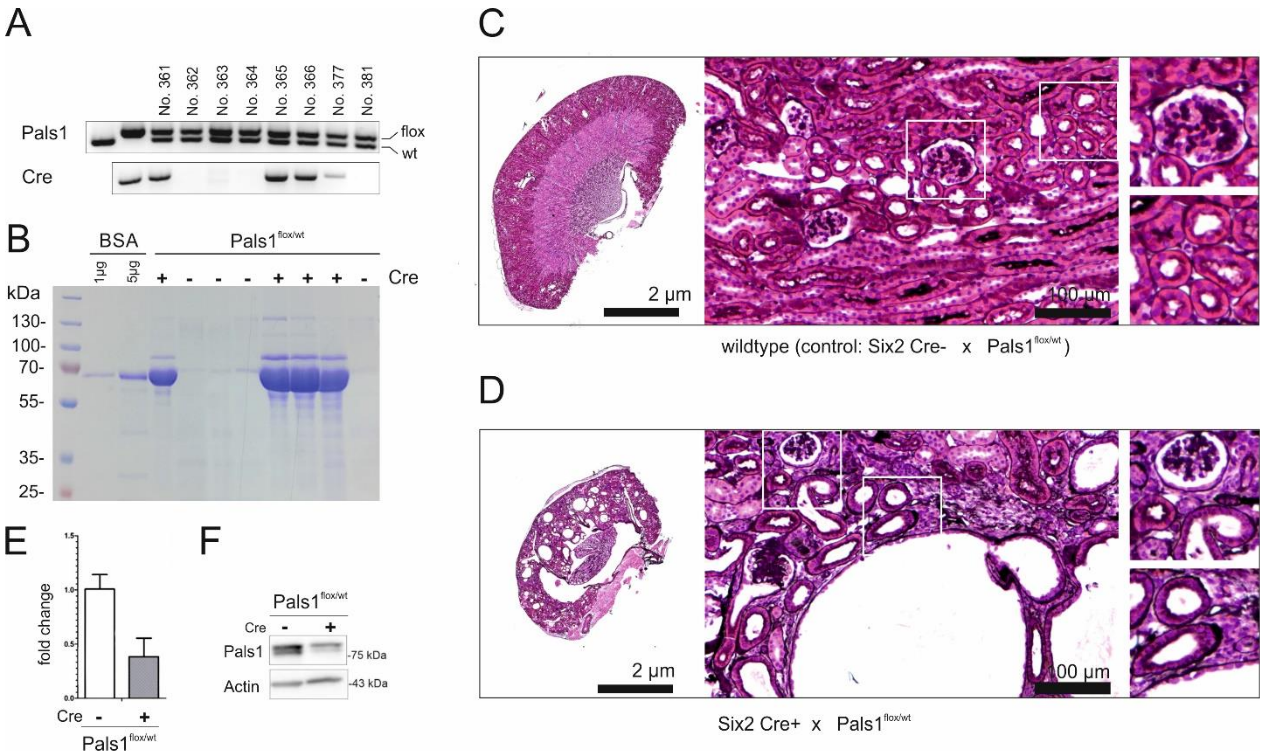

SM10: *Data set: Used Pals1-deficient kidney specimen*

Breeding of homozygous Pals1$^{flox/flox}$ mice with heterozygous Six2-Cre transgenic mice results in the 50% of Pals1$^{flox/wt}$ X Six2-Cre (Cre+) and 50% Pals1$^{flox/wt}$ (Cre-) mice. All of Pals1-haplodeficient mice show a full penetrant phenotype that develops heavy proteinuria and renal cysts. **(A)** Agarose gel showing the heterozygosity of used mice for the Pals1 allele (animal No. 361-365, and 377 and 381; flox: floxed allele, wt: wildtype, Cre positive genotyping). **(B)** SDS-polyacrylaminde gel analysis demonstrated that only Cre+ mice develop proteinuria. **(C/D)** Histologic analyses (Jones staining) of kidney sections. Wildtype mice (C; Cre-) show normal kidney morphology (left: overview, right: details). In contrast, Pals1-haplodeficient (Cre+) kidneys show very large cysts and numerous dilated tubules (D; Cre+ left: overview, right: details). The Pals1 expression level in kidneys of Pals1$^{flox/wt}$ X Six2-Cre (Six2-Cre+) and their Pals1$^{flox/wt}$ littermate controls (Six2-Cre-) was analyzed on mRNA **(E)** and protein level **(F)**. Both approaches confirm a downregulation of Pals1 expression of approximately 50%.

METHODS

The experimental protocols and methods in this work involving animals were approved by and conducted in accordance with all guidelines and regulations set forth by the German regional authorities (Az.: 84–02.04.2014 A405; LANUV). Animals were housed under standard specific pathogen–free conditions with free access to tap water and standard animal chow. Pals1 conditional knockout and Six2-Cre transgenic mice, their genotyping PCRs and primers (Table ST8) have been described earlier (Kobayashi et al., 2008; Kim et al., 2010; Weide et al., 2017).

Methods used (for experiments shown in suppl. data set A, including SDS-PAGE, genotyping of mice and analyses of histologic section) have been described earlier in detail (Weide et al., 2017)

References

Kim, S., Lehtinen, M. K., Sessa, A., Zappaterra, M. W., Cho, S. H., Gonzalez, D., et al. (2010). The Apical Complex Couples Cell Fate and Cell Survival to Cerebral Cortical Development. *Neuron* 66, 69–84. doi:10.1016/j.neuron.2010.03.019.

Kobayashi, A., Valerius, M. T., Mugford, J. W., Carroll, T. J., Self, M., Oliver, G., et al. (2008). Six2 Defines and Regulates a Multipotent Self-Renewing Nephron Progenitor Population throughout Mammalian Kidney Development. *Cell Stem Cell* 3, 169–181. doi:10.1016/j.stem.2008.05.020.

Weide, T., Vollenbröker, B., Schulze, U., Djuric, I., Edeling, M., Bonse, J., et al. (2017). Pals1 Haploinsufficiency Results in Proteinuria and Cyst Formation. *J. Am. Soc. Nephrol.* doi:10.1681/ASN.2016040474.

SM11 Data Set*:* Immunohistochemical analyses using antibodies against Slc5a2, Slc22a7 and Slc22a8

The transcriptome analyses show that Pals1 gene silencing in mouse induce downregulation of various renal mRNA transporters of the SLC family including Slc5a2 (Sglt2), or Slc22a7 (Oat2), and a trend for Slc22a8 (Oat3). In Pals1-deficient kidneys (Cre+), most of the proximal convoluted tubules (PCT; S1/S2 segments) as well proximal straight tubules (PST; S3 segments) were significantly dilated in comparison to those in the littermate wildtype controls (Cre -). Also, our immunohistochemical (IHC) data indicate that cysts could possibly originate from both proximal and distal nephron segments. Although the proximal tubules are dilated, basolateral and apical membrane domain of their epithelium look morphologically preserved.

Tested downregulated SLC transporters were mainly located in the BBM (cortical S1/S2 segments for Slc5a2/Sglt2 and S3 segments in the outer stripe for Slc22a7/Oat2), indicating that Pals1 might has an impact on SLC transporters located in the apical membrane domain of proximal tubule epithelium in contrast to less sensitive SLC transporters located in the basolateral membrane domain. The Na/K-ATPase-related staining was used to label the basolateral membrane domain of proximal and distal tubules of mouse nephrons.

These labelings confirm data shown in Fig. 2C, indicating that polarity of proximal tubules is preserved in the Pals1-haplodeficient kidneys. Furthermore, tested renal SLC membrane transporters are located on the same epithelial domain of proximal tubules in both Pals1-depleted and wildtype epithelia confirming the preservation of cell polarization in Pals1-haplodeficient nephrons. In addition to the staining given in Fig. 2C, we performed further IHC using specific antibodies against Slc5a2 (Sglt2), Slc22a7 (Oat2), and Slc22a8 (Oat3) as well as Na/K-ATPase (α1-subunit).

SM11-a: Impact of Pals1 silencing on the Slc5a2 (Sglt2) localization in the mouse kidney
SM11-b: Localization of Slc5a2 (Sglt2) in the kidney of control (wildtype) animals; rats vs. mice

SM11-c: Impact of Pals1 silencing on the Slc22a7 (Oat2) localization in the mouse kidney
SM11-d: Localization of Slc22a7 (Oat2) in the kidney of control (wildtype) animals; rats vs. mice
SM11-e: Double labeling of Slc22a7 (Oat2) and Na/K-ATPase (α1-subunit) in the mouse kidney; wildtype vs. Pals1 deficient
SM11-f: Impact of Pals1 silencing on the Slc22a8 (Oat3) localization in the mouse kidney
SM11-g: Localization of Slc22a8 (Oat3) in the kidney of control (wildtype) mice

SM11-h: Impact of Pals1 silencing on the Na/K-ATPAse (α1-subunit) localization in mouse kidney

Slc5a2

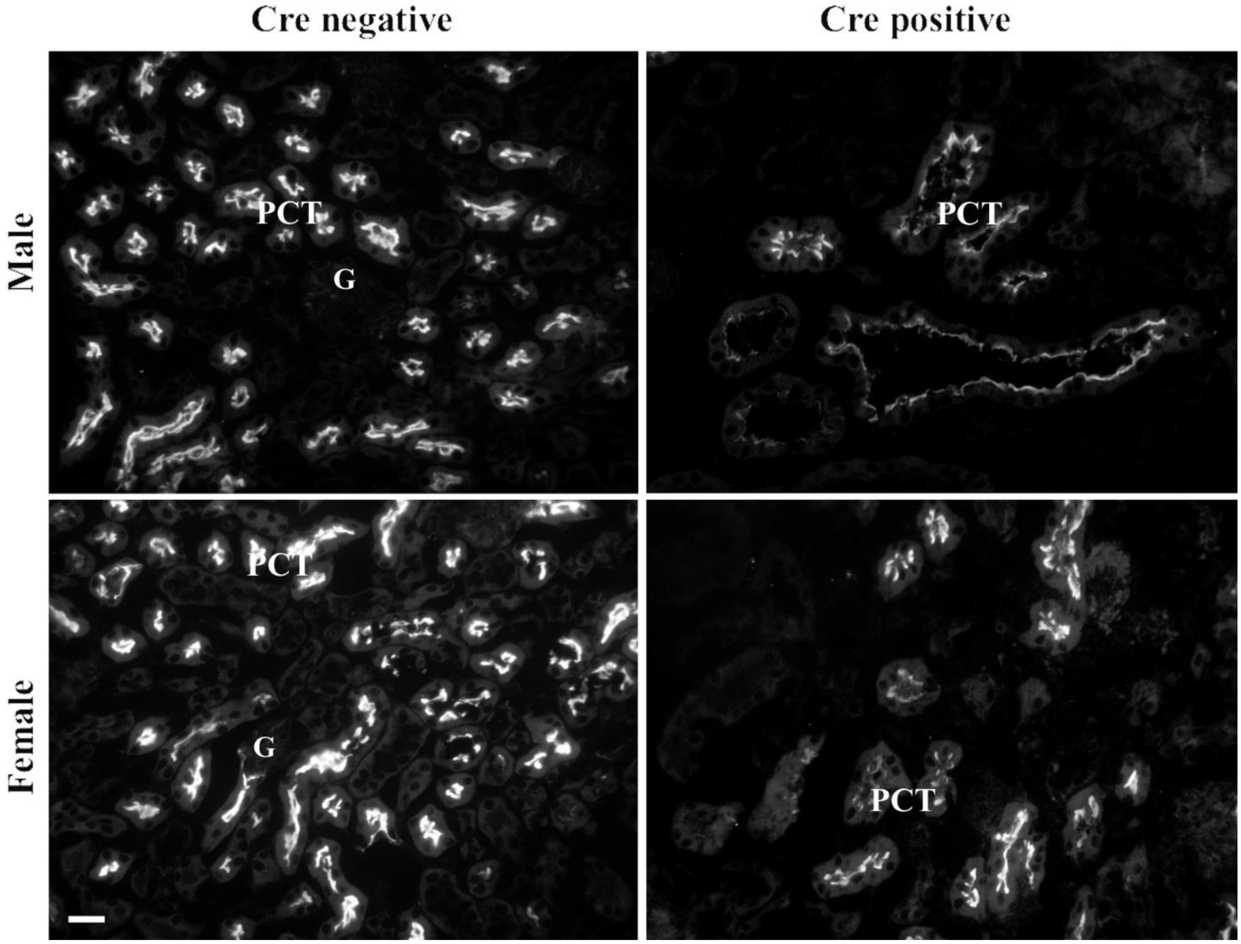

SM11-a: Impact of Pals1 silencing on the Slc5a2 (Sglt2) localization in the mouse kidney

In the kidney of Cre negative wildtype mice (littermate control), the anti Slc5a2/Sglt2 antibody strongly stained the brush border membrane (BBM) of proximal convoluted tubules (PCT, S1/S2 segments) in the cortex where the Slc5a2-related fluorescence intensity was similar in males and females. In the kidney of Pals1-deficient (Cre positive) mice, the anti Slc5a2/Sglt2 antibody stained the same tubular epithelial domain, i.e. the BBM of PCT S1/S2 segments. However, fluorescence intensity of Slc5a2 BBM staining of PCT in the kidney of Pals1-deficient (Cre positive) mice was lower in comparison to wildtype (Cre positive) mice; this phenomenon was observed in both male and female mice. In the kidney of Pals1-deficient (Cre positive) mice almost all PCT was significantly dilated in comparison to PCT of wildtype Cre negative mice. Furthermore, in the kidney of Pals1-deficient (Cre positive) mice, the presence of many cysts was detected (not shown). Bar = 20 µm.

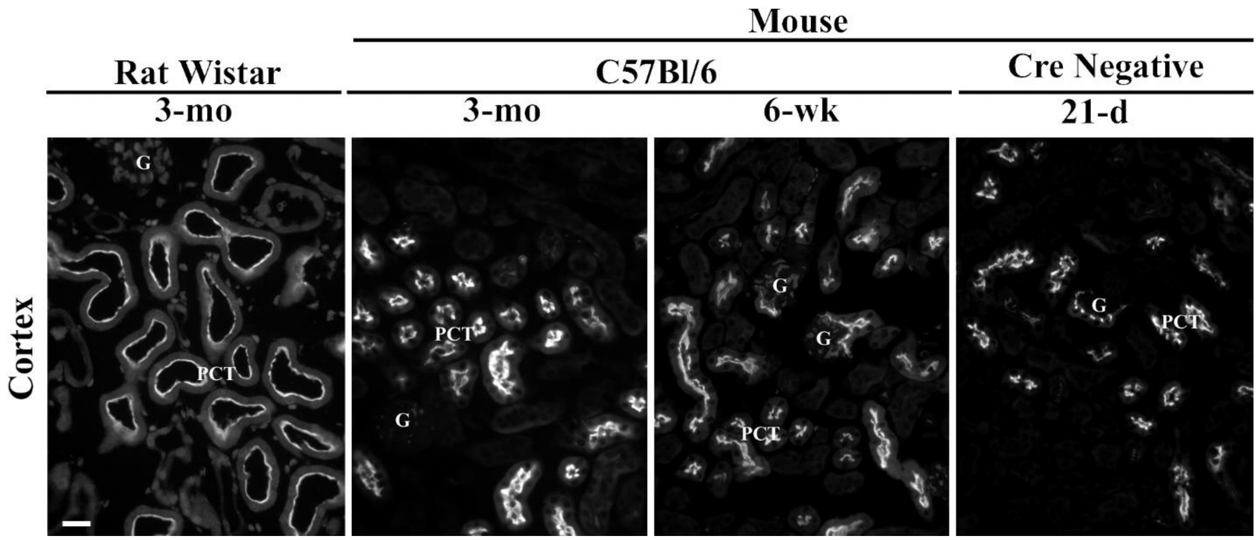

SM11-b: Localization of Slc5a2 (Sglt2) in the kidney of control (wildtype) animals; rats vs. mice

In the kidney of 3-months old rats, the Slc5a2/Sglt2 antibody strongly stained the brush border membrane (BBM) of proximal convoluted tubules (PCT, S1/S2 segments) in the cortex. In the kidney of 3-months/6-weeks old C57Bl/6 mice, the Slc5a/Sglt2 antibody strongly stained the BBM of PCT (S1/S2 segments), whereas other nephron segments were Slc5a2-negative. These results are in accordance to previously reported data (Sabolić et al., 2012). The same pattern of Sglt2-related immunostaining was observed in the kidney of 21-d old wildtype (Cre Negative) mice. G, glomeruli. Bar = 20 µm.

SLC22a7

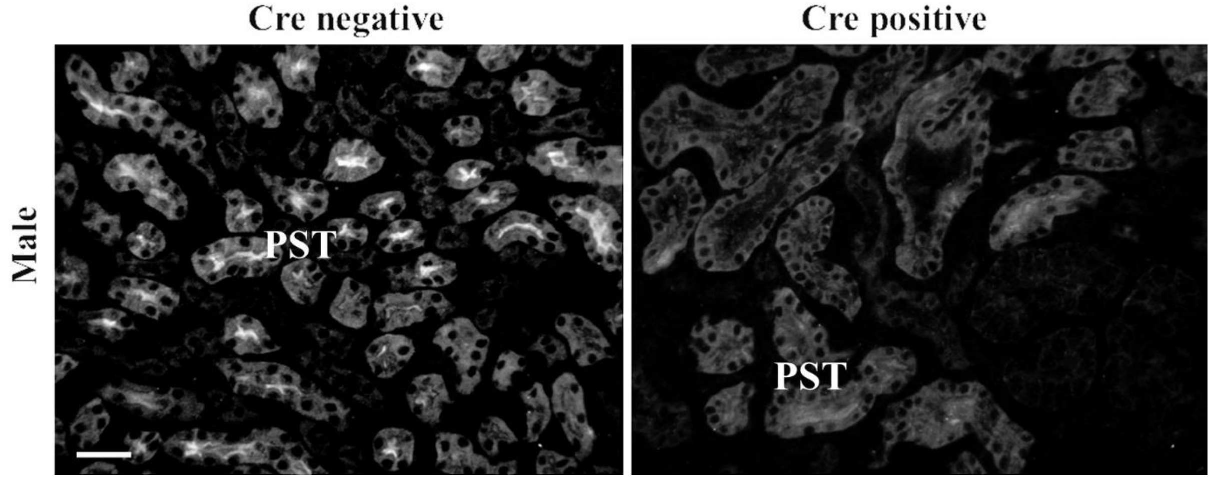

SM11-c: Impact of Pals1 silencing on the Slc22a7 (Oat2) localization in the mouse kidney

In the kidney of 21 day old wildtype (Cre negative) mice, the Slc22a7/Oat2-antibody strongly stained the brush border membrane (BBM) of proximal straight tubules (PST) (S3 segments) in the outer stripe where the Slc22a7/Oat2-related fluorescence intensity was similar in males and females (data not shown). In the kidney of Pals1-deficient (Cre positive) mice, the Slc22a7/Oat2 antibody faintly stained the same tubular epithelial domain, i.e. BBM of PST S3 segments. In other words: fluorescence intensity of Slc22a7/Oat2-related BBM staining of PST S3 segments in the kidney of Pals1 depleted epithelia was drastically lower in comparison to the controls (Cre negative). Also, in the kidney of Pals1-deficient (Cre positive) mice almost all PST S3 segments in outer stripe were dilated in comparison to PST S3 segments of wildtype (Cre negative) mice. Furthermore, in the kidney of Pals1-deficient (Cre positive) mice, presence of many cysts was detected (not shown). Bar = 20 µm.

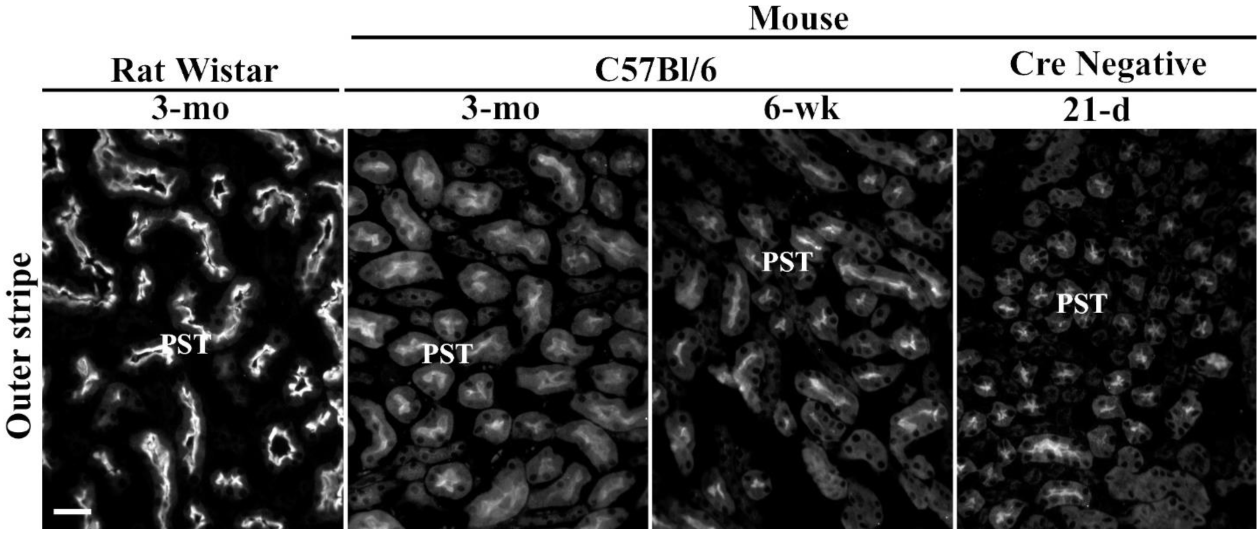

SM11-d: Localization of Slc22a7 (Oat2) in the kidney of control (wildtype) animals; rats vs. mice

In the kidney of 3 months (mo) old rat, the Slc22a7/Oat2 antibody strongly stained the brush border membrane (BBM) of proximal straight tubules (PST, S3 segments) in the outer stripe. In the kidney of 3-mo/6-weeks (wk) old C57Bl/6 mice, the Slc22a7/Oat2 antibody strongly stained the BBM of PST S3 segments in the outer stripe, whereas other nephron segments were Oat2-negative. These results are in accordance to our previously reported data and were described in detail (Ljubojevic et al., 2007). The same pattern of Slc22a7/Oat2 -related immunostaining was observed in the kidney of 21 days old wildtype (Cre negative) mice. Bar = 20 µm.

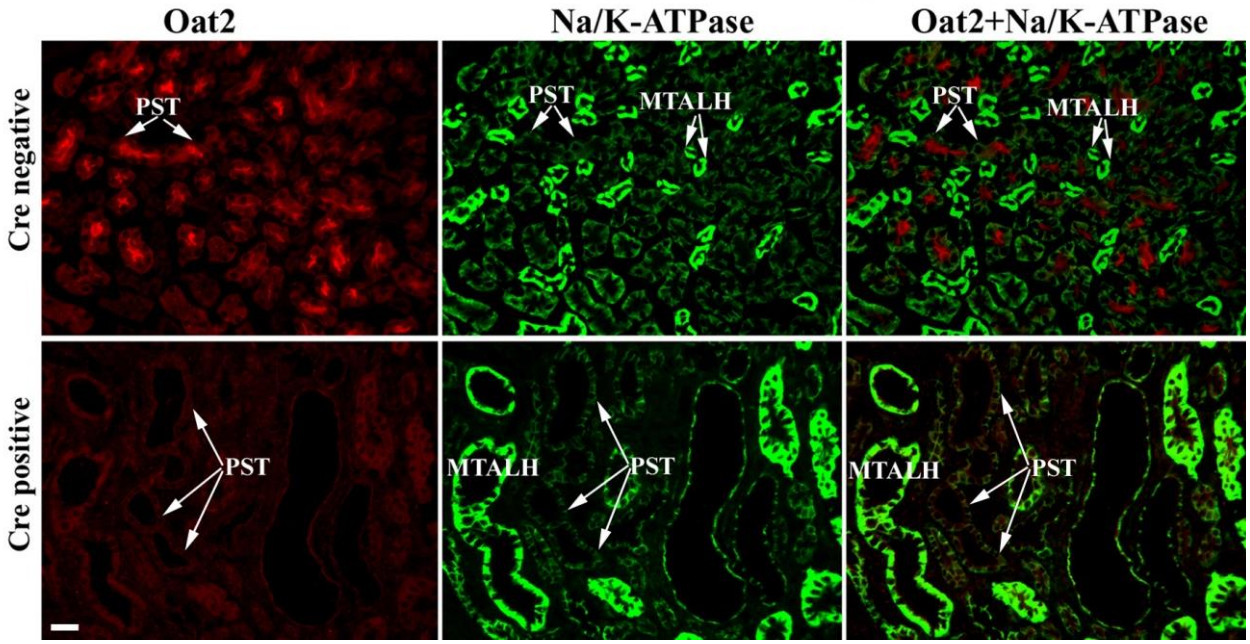

SM11-e: Double labeling of Slc22a7 (Oat2) and Na/K-ATPase (α1-subunit) in the mouse kidney; wildtype vs. Pals1-deficient

In the kidney of wildtype (Cre negative) mice, the Slc22a7/Oat2 antibody (red) strongly stained the brush border membrane (BBM) of proximal straight tubules (PST) (S3 segments) in the outer stripe, whereas fluorescence intensity of this immunostaining was significantly reduced in the kidney of Pals1-deficient (Cre positive) mice. In the outer stripe of wildtype (Cre negative) and Pals1-deficient (Cre positive) mice, the Na/K-ATPase-antibody (green) stained the BLM of PST as well the medullary thick ascending limb of Henle (MTALH). However, in the outer stripe of Pals1-deficient (Cre positive) mice most of the PST S3 segments were dilated, and presence of many cysts was detected. Merged imaged (red + green fluorescence) indicated that Oat2-related immunostaining was almost undetectable in the PST S3 segments of Pals1-deficient (Cre positive) mice in comparison to wildtype (Cre negative) mice.
Bar = 20 µm.

Slc22a8

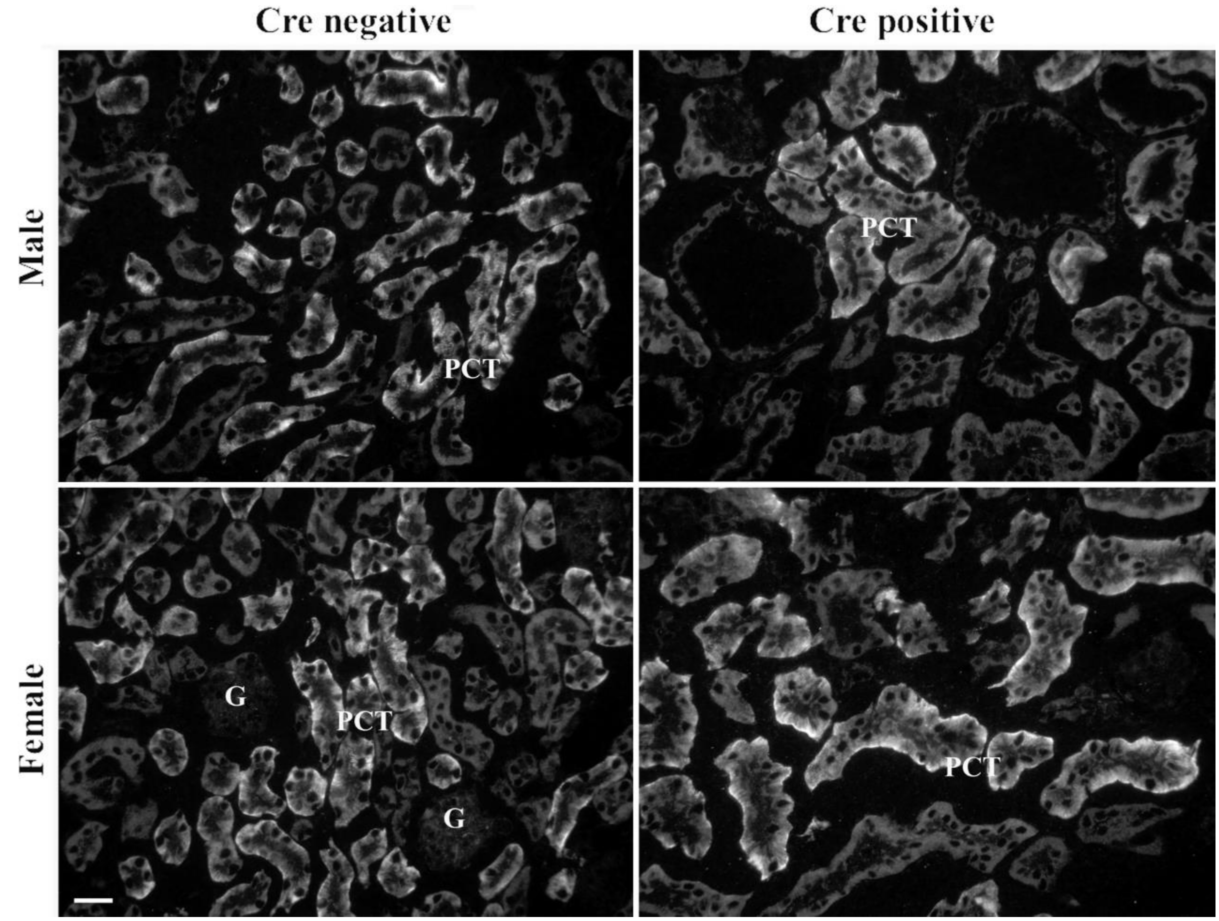

SM11-f: Impact of Pals1 silencing on the Slc22a8 (Oat3) localization in the mouse kidney

In the kidney of 21 days old wildtype (Cre negative) mice, the Slc22a8/Oat3-Ab strongly stained the basolateral membrane (BLM) of proximal convoluted tubules (PCT) in the cortex where the Oat3-related fluorescence intensity was similar in males (M) and females (F). In the kidney of *Pals1*-deficient (Cre positive) mice, the Slc22a8/Oat3-Ab strongly stained the same tubular epithelial domain, i.e. BLM of cortical PCT. Importantly, in the *Pals1*-deficient (Cre positive) mice, almost all Oat3-positive PCT were significantly dilated in comparison to Oat3-positive PCT of wildtype (Cre negative) mice; extent of PCT dilatation in the kidneys of *Pals1*-deficient (Cre positive) animals was similar in M and F. However, fluorescence intensity of Oat3-related BLM staining of cortical PCT was similar in kidneys of wildtype and *Pals1*-deficient mice. Furthermore, in the kidney of *Pals1*-deficient (Cre positive) mice, presence of many cysts was detected (not shown). G, glomeruli. Bar = 20 µm

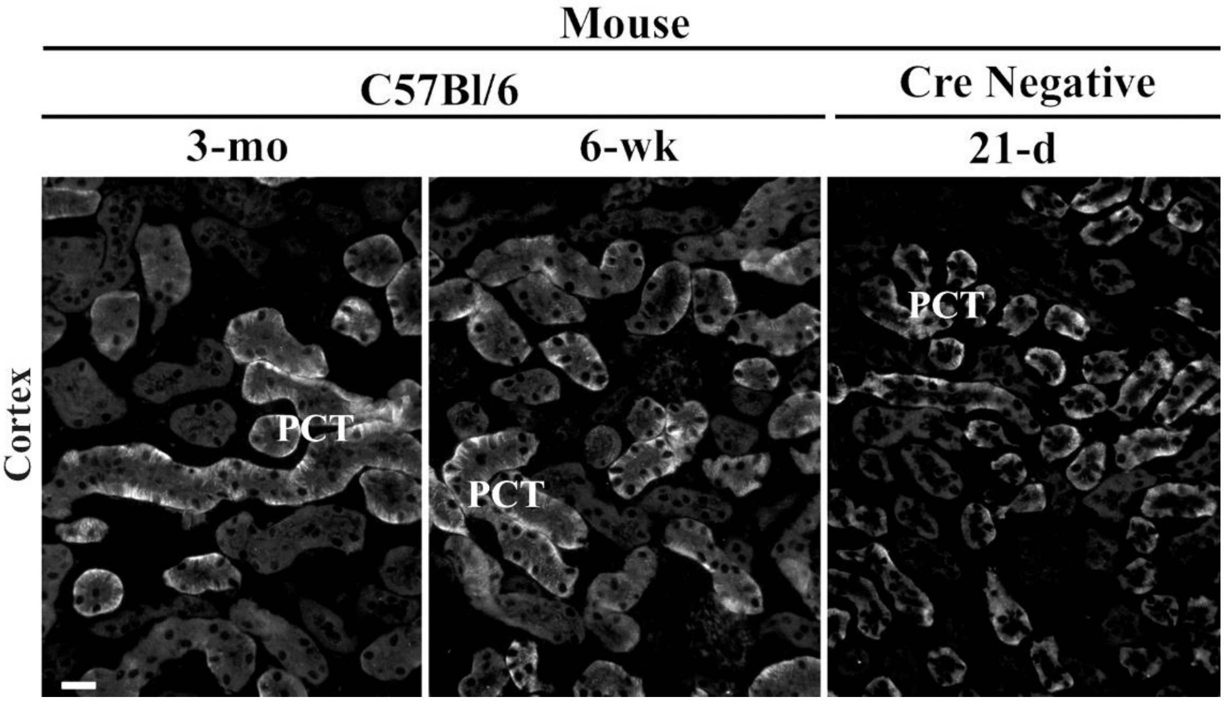

SM11-g: Localization of Slc22a8 (Oat3) in the kidney of control (wildtype) mice

In the kidney of 3-months/6-weeks old C57Bl/6 mice, the Slc22a8/Oat3 antibody stained the basolateral membrane of proximal convoluted tubules (PCT, mainly S2 segments) in the cortex, whereas other nephron segments were Slc22a8/Oat3-negative. These results are in accordance to our previously reported data and were described in detail previously (Breljak et al., 2013). The same pattern of Slc22a8/Oat3-related immunostaining was observed in the kidney of 21 days old wildtype (Cre negative) mice. Bar = 20 µm.

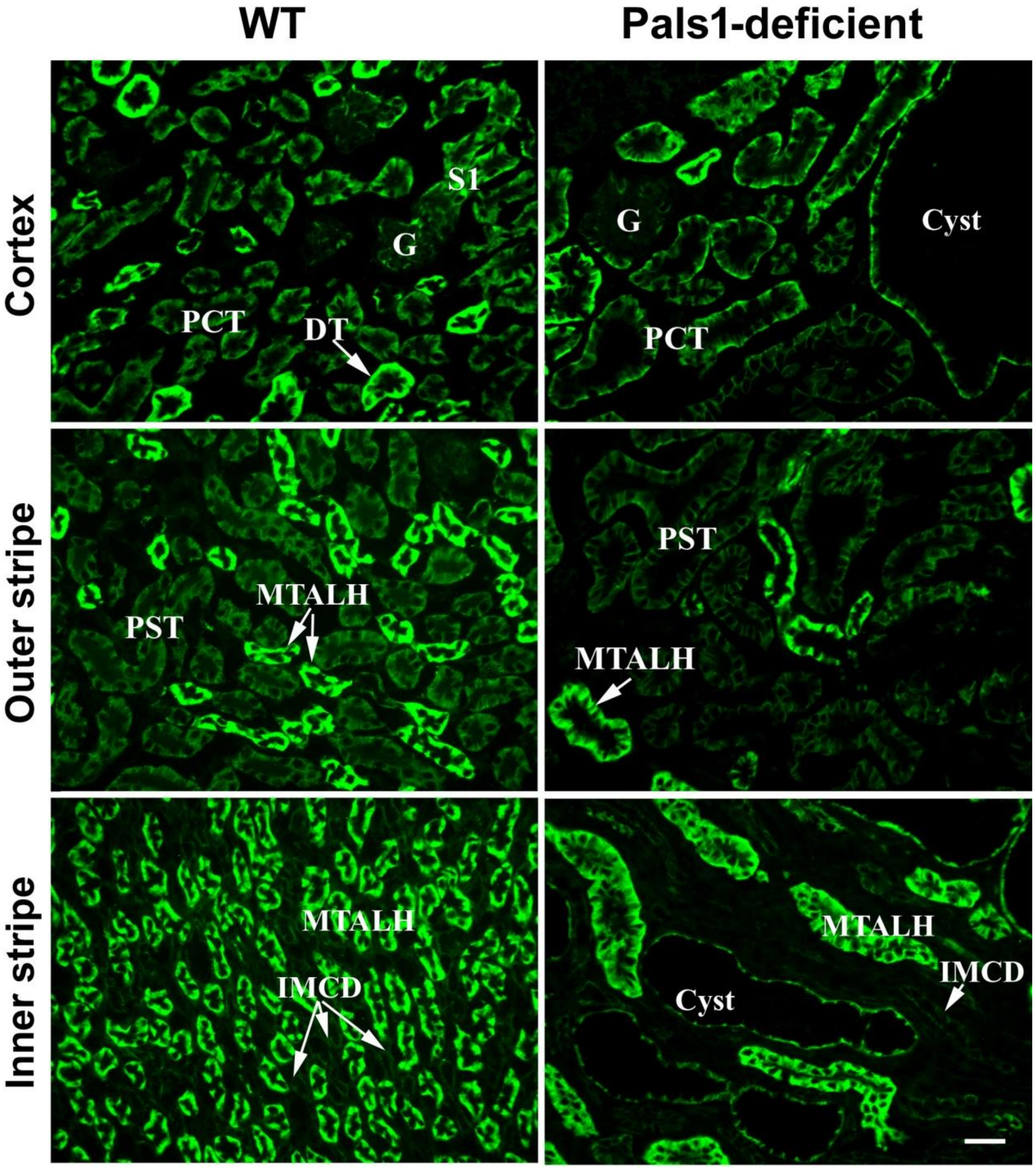

SM11-h: Impact of Pals1 silencing on the Na/K-ATPAse (α1-subunit) localization in mouse kidney

In the kidney of WT mice, the Na/K-ATPase antibody stained with heterogeneous intensity the basolateral membrane (BLM) of proximal convoluted tubules (PCT) in the cortex and the BLM of proximal straight tubules (PST) in the outer stripe as well as distal nephron segments including distal tubules (DT), cortical thick ascending limb of Henle (CTALH) (not shown), medullary thick ascending limb of Henle (MTALH) and collecting ducts (CD) along the entire nephron including cortical collecting ducts (CCD), outer medullary collecting ducts (OMCD) (both not shown) and inner medullary collecting ducts (IMCD). In the kidney of Pals1-deficient mice, the Na/K-ATPase antibody stained the same membrane domain (BLM) along the entire nephron. However, in the Pals1-deficient mice, most of the tubules are dilated; some of them are extremely dilated. Na-K-ATPase-related staining indicated that these cysts possible originated from both proximal nephron segments (PCT and PST) as well as distal nephron segments (TALH and CD).

Bar = 20 µm

MATERIAL AND METHODS

Antibodies: The antibodies for the SLCs family members including organic anion transporters (Oat) and sodium-glucose cotransporter 2 (Sglt2) used in this study, have been described in detail previously (Ljubojevic et al., 2007.; Sabolic et al., 2012., Breljak et al., 2013). Commercial monoclonal antibodies for the Na/K-ATPase α1-subunit (sc-48345) and β-Actin (sc-47778) were purchased from Santa Cruz Biotechnology, Inc. (Santa Cruz, CA, USA) and their use was described previously (Breljak et al., 2016). Commercial secondary antibodies CY3-labeled goat anti-rabbit IgG (GAR-CY3) and fluorescein isothiocyanate-labeled donkey anti-mouse IgG (DAM-FITC) were purchased from Jackson Immuno Research Laboratories Inc. (West Grove, PA, USA).

Chemicals: Chemicals and reagents for tissue fixation and immunocytochemistry were the analytical or molecular biology grade and their commercial source was Kemika (Zagreb, Croatia), Sigma (St. Louis, MO, USA) or Fisher Scientific (Pittsburgh, PA, USA).

Tissue fixation and immunofluorescence microscopy: Mouse kidney tissues were fixed overnight in 4% *p*-formaldehyde. After fixation, tissue pieces were washed and stored in PBS (+0.02% NaN3) until used. Before cutting cryosections, tissue pieces were infiltrated with 30% sucrose (in PBS) overnight, embedded in tissue freezing medium Compound 4583 O.C.T. (Vector Labs, USA), frozen at 25°C, and sectioned with a Leica CM 1850 cryostat (Leica instruments, Nussloch, Germany). Four µm thick cryosections were collected on Superfrost/Plus microscope slides (Thermo Scientific, Germany), dried at room temperature for a few hours, and stored at 4°C until use. In order to reveal of antibody binding sites, antigen retrieval technique was applied as described previously in detail (Brzica et al., 2009). Briefly, frozen tissue sections were heated in a microwave oven in 10 mM citrate buffer at pH 6, treated with Triton X-100 containing buffers, blocked with 1% bovine serum albumin before applying primary antibodies diluted in PBS for Oat2/Slc22a7 (1:500), Oat3/Slc22a8 (1:100) and Sglt2/Slc5a2 (1:2000); and secondary antibody: GAR-CY3 (1:800). The stained sections were examined and photographed with an Opton III RS fluorescence microscope (Opton Feintechnik, Oberkochen, Germany) using a Spot RT Slider digital camera and software (Diagnostic Instruments, Sterling Heights, MI, USA). Immunostaining was inspected under the specific filter for red (CY3-related) and green (FITC-related) fluorescence. Images presented representative immunohistochemical analysis of kidney cryosections from 5 independent males (Pals1-deficient vs. wildtype) and 1 females (Pals1-deficient vs. wildtype), and were acquired at the identical microscope and camera settings. The photos were imported into Adobe Photoshop 6.0 software for processing,

assembling and labelling. The same software was used for conversion of the CY3-related red fluorescence into black and white mode. To perform double staining of Oat2/Slc22a7 and Na/K-ATPase, the kidney cryosections were first stained for Oat2/Slc22a7 as described above, and then incubated with Na/K-ATPase antibody (1:100) at 4°C overnight, washed and incubated with DAM-FITC (1:50) at room temperature for 60 min. The stained sections were examined, and the images were collected as described above. The images with CY3-related red and FITC-related green fluorescence were imported, merged and processed in Adobe Photoshop.

References

Breljak, D., Brzica, H., Sweet, D. H., Anzai, N., and Sabolic, I. (2013). Sex-dependent expression of Oat3 (Slc22a8) and Oat1 (Slc22a6) proteins in murine kidneys. *Am. J. Physiol. - Ren. Physiol.* 304. doi:10.1152/ajprenal.00201.2012.

Breljak, D., Ljubojević, M., Hagos, Y., Micek, V., Balen Eror, D., Vrhovac Madunić, I., et al. (2016). Distribution of organic anion transporters NaDC3 and OAT1-3 along the human nephron. *Am. J. Physiol. - Ren. Physiol.* 311. doi:10.1152/ajprenal.00113.2016.

Brzica, H., Breljak, D., Ljubojević, M., Balen, D., Micek, V., Anzai, N., et al. (2009). Optimal methods of antigen retrieval for organic anion transporters in cryosections of the rat kidney. *Arh. Hig. Rada Toksikol.* 60. doi:10.2478/10004-1254-60-2009-1895.

Ljubojević, M., Balen, D., Breljak, D., Kušan, M., Anzai, N., Bahn, A., et al. (2007). Renal expression of organic anion transporter OAT2 in rats and mice is regulated by sex hormones. *Am. J. Physiol. - Ren. Physiol.* 292. doi:10.1152/ajprenal.00207.2006.

Sabolić, I., Vrhovac, I., Eror, D. B., Gerasimova, M., Rose, M., Breljak, D., et al. (2012). Expression of Na +-D-glucose cotransporter SGLT2 in rodents is kidney- specific and exhibits sex and species differences. *Am. J. Physiol. - Cell Physiol.* 302. doi:10.1152/ajpcell.00450.2011.
